# Bibliometric analysis of ferroptosis: a comprehensive evaluation of its contribution to cancer immunity and immunotherapy

**DOI:** 10.3389/fonc.2023.1183405

**Published:** 2023-04-27

**Authors:** Zhen Wang, Hui Zhang, Li Wang, Zhen Ma, Yu’ang Cui, Haitian Fu, Chunjing Yu

**Affiliations:** ^1^ Wuxi School of Medicine, Jiangnan University, Wuxi, China; ^2^ Department of Nuclear Medicine, Affiliated Hospital of Jiangnan University, Wuxi, China

**Keywords:** ferroptosis, immunotherapy, anti-tumor immunity, nanoparticles, gene signature, bibliometric analysis

## Abstract

**Background:**

In the past 5 years, ferroptosis-associated cancer immunity has been attracted significant research interest.

**Objective:**

This study was performed to identify and analyze the global output trend for ferroptosis in cancer immunity.

**Methods:**

Relevant studies were retrieved from the Web of Science Core Collection on Feb 10^th^, 2023. The VOSviewer and Histcite softwares were utilized to perform the visual bibliometric and deep mining analyses.

**Results:**

A total of 694 studies (530 articles (76.4%) and 164 (23.6%) review articles) were retrieved from the Web of Science Core Collection for visualization analyses. The top 3 key keywords were ferroptosis, prognosis and immunotherapy. The top 30 local citation score (LCS) authors were all collaborators of Zou Weiping. Deep mining of 51 nanoparticle-related articles showed that BIOMATERIALS was the most popular journal. The primary goal of gene signatures related to ferroptosis and cancer immunity was to establish prognostic predictions.

**Conclusion:**

There has been a significant increase in ferroptosis-associated immune publications in the recent 3 years. The key research hotspots include mechanisms, prediction and therapeutic outcomes. The most influential article was from the Zou Weiping’s group, which proposed that system xc-mediated ferroptosis is induced by CD8(+) T cell-secreted IFNγ after PD-L1 blockage for immunotherapy. The frontier of research in the field of ferroptosis-associated immune is the study on nanoparticle and gene signature The limitation of this bibliometric study is that publications on this topic are few.

## 1 Introduction

Cancer is a malignant tumor. People are more likely to die from malignant tumors as their incidences have risen significantly in recent years. According to the latest cancer epidemiological data, nearly 10 million people died of cancer in 2020, accounting for nearly one in six deaths, which highlights the serious threat the disease imposes on public health (https://www.who.int/news-room/fact-sheets/detail/cancer). Cancer can be treated in various ways, and current strategies involve a combination of surgery, radiotherapy, chemotherapy, immunotherapy, and other methods (1). Among these strategies, immunotherapies are the most promising. Major immunotherapy approaches include inhibitory immune checkpoints (ICB) blockade, antigen-specific peptide vaccination, oncolytic virotherapy, and adoptive cell therapies, and they are based on the key role of tumor-specific T cell activation (2). Immunogenic cell death (ICD) caused by immunotherapy results in long-lasting and effective immunological memory compared to other types of cell death (3–5).

However, many cancer cells enable to develop immune escape and relapse pathways, so some cancer types cannot respond to effective primary immunotherapy (6). Besides immunotherapy triggered ICD, ferroptosis is another type of immunogenic cell death (7). A study by Dmitri V Krysko et al. showed that early ferroptotic tumor cells stimulated tumor immunity, produced immune memory, and persistently killed cancer cells (8). Ferroptosis was first proposed and named by Stockwell BR’s group, which pioneered studies on ferroptosis (9). Canonical ferroptosis involves the suppression of system xc−, glutathione (GSH), and glutathione peroxidase 4 (GPX4), which accumulates reactive oxygen species (ROS), phospholipid peroxidation (PUFA-PL), and iron (10, 11). Additionally, Acyl-CoA Synthetase Long-Chain Family Member 4 (ACSL4) is a promoter that executes ferroptosis. High ACSL4 expression or activity sensitize and promote cell ferroptosis (12, 13). In addition, targeting ferroptosis is considered to be an effective cancer treatment strategy. Most of the treatment strategies are based on the mechanism of ferroptosis are aimed at identifying sensitive signature panels of ferroptosis and improve the efficacy of anti-cancer therapies. Numerous studies have reported that ferroptosis inducers are sensitive to many cancers such as glioma, triple negative breast cancer (14, 15) and provide more opportunities for the treatment of cancer (16).

Early in 2018, Jennifer Tsoi et al. first showed that ferroptosis-inducing agents are sensitive to reduced dedifferentiated melanoma cells after induction of Interferon Gamma (IFNγ) (17). Subsequently, Zou’s group found that the ovarian cancer cells killed by immunotherapy PD-L1 blockage were ferroptotic, and the mechanism underlying this process involved the function of IFNγ released by PD-L1 blockage-activated CD8 (+) T cells. IFNγ was able to target cancer cells by suppressing system Xc^-^ and lead to ferroptosis as a result (18). In addition, combining immunotherapy and radiotherapy followed the same process (19). However, combining IFNγ and AA triggered ACSL4-induced intrinsic ferroptosis instead (20).

**Figure 1:**
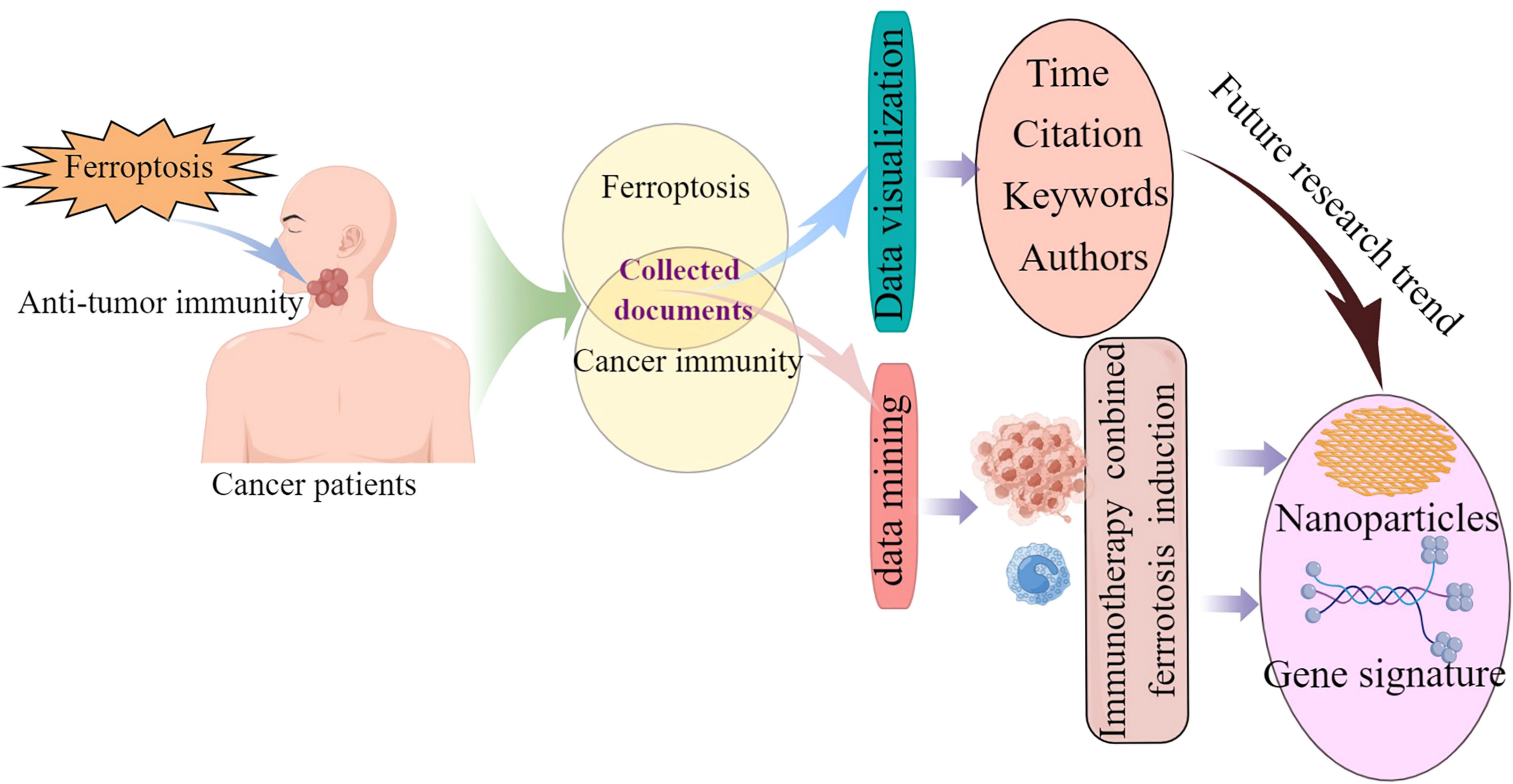
The graphic abstract of this research. The detailed description is in abstract.

In this study, original articles and review articles on ferroptosis in cancer immunity were identified from the Web of Science Core Collection using VOSviewer. Histcite was used to visualize and analyze the collected articles. This bibliometric study identifies and analyzes the global trend on ferroptosis in cancer immunity. The graphic abstract is shown in **Figure 1**. Our analysis is expected to inspire researchers and provide reference data to improve future management of scientific work.

## 2 Materials and methods

### 2.1 Search strategy and data collections

**Figure 2:**
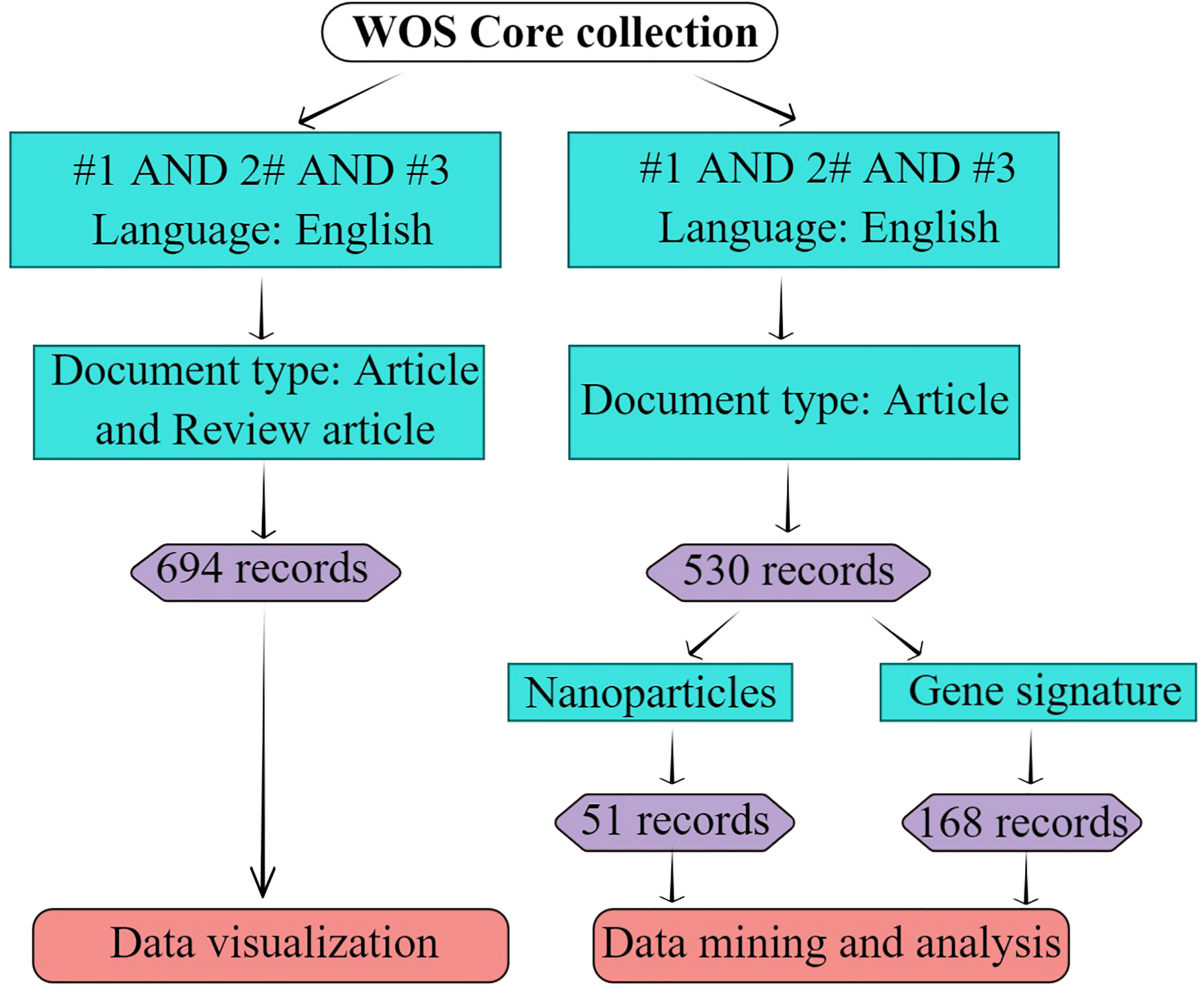
Search strategy. #1 represents “(((((((((TS=(anti-tumor immunity)) OR TS=(immunity)) OR TS=(cancer immunotherapy*)) OR TS=(anti-tumor immunotherapy*)) OR TS=(immune therapy*)) OR TS=(anti-tumor immune therapy*)) OR TS=(immunology therapy*)) OR TS=(anti-tumor immunology therapy*)) OR TS=(Radioimmunotherapy*)) OR TS=(Immunomodulation)”; #2 represents “TS=(Neoplasm* or cancer$)”; #3 represents “TS=(ferropto*)”; “*” represents infinite truncator; “$” represents finite truncator; the retrieval date was Feb 10^th^, 2023. This figure was drawn by Figdraw of HOME for Researchers.

Bibliometric data for this study were collected from the Web of Science Core Collection, including Science Citation Index Expanded (SCI-EXPANDED), Social Sciences Citation Index (SSCI), Arts & Humanities Citation Index (AHCI), Emerging Sources Citation Index (ESCI) from, Current Chemical Reactions (CCR-EXPANDED), and Index Chemicus (IC). The terms ‘ferroptosis,’ ‘immunity,’ and ‘cancer’ were searched in the MeSH (https://www.ncbi.nlm.nih.gov/mesh). The words or phrases, such as anti-tumor immunity, immunity, tumor immunotherapy, anti-tumor immunotherapies, anti-tumor immune therapy, immune therapy, immunology therapy, anti-tumor immunology therapy, radioimmunotherapy, immunomodulation, and Neoplasm were used in the documents of cancer immunity field. Ferroptotic, cancer(s), and Neoplasm(s) were also applied in some documents. Truncators were adopted to avoid missing documents during searching and to obtain more comprehensive data. *” represented an infinite truncated word, and $ represented a finite truncated word. To obtain a systematic and comprehensive analysis of ferroptosis in the cancer immunity field, the following search strategy was applied; #1 represented (((((((((TS = (anti-tumor immunity)) OR TS = (immunity)) OR TS = (cancer immunotherapy*)) OR TS = (anti-tumor immunotherapy*)) OR TS = (immune therapy*)) OR TS = (anti-tumor immune therapy*)) OR TS = (immunology therapy*)) OR TS = (anti-tumor immunology therapy*)) OR TS = (Radioimmunotherapy*)) OR TS = (Immunomodulation), #2 represented TS = (Neoplasm* or cancer$), and #3 represented TS = (ferropto*). The final search strategy is TS = **#**1 AND #2 AND #3. Only articles published in English and full articles and reviews were considered. Other document types, including early access, editorial materials, meeting abstracts, corrections, and book chapters, were excluded. The search flow chart is shown in **Figure 2**. The search was completed on Feb 10^th^, 2023.

### 2.2 Methodology

Bibliometric visualization and deep mining analysis were performed using VOSviewer (VOSviewer version 1.6.18) and HistCite (HistCite Pro 2.1). The distribution of publication years, the ratio of article and review articles, and the statistics of cancer-type were determined using Microsoft Excel 2016.

## 3 Results

### 3.1 Years and cancer-type distribution involved in the collected publication documents

**Figure 3:**
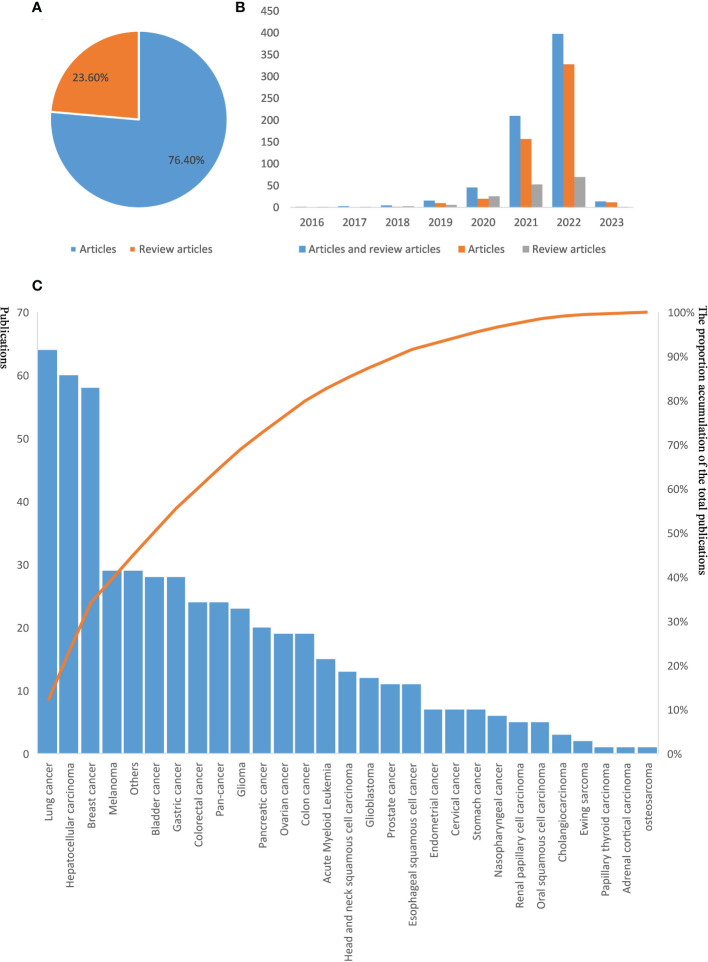
The distribution among years, article type, and cancer type. **(A)** the proportion of articles and reviews. **(B)** abscissa axis on the publication year. The vertical coordinate represents the number of published documents, the blue column represents the sum of the article and reviews article documents, the orange column represents article documents, the gray column represents review article documents. **(C)** distribution of involved cancer type.

A total of 530 (76.4%) articles (after removing one duplicate from 531) and 124 (23.6%) reviews were retrieved (**Figure 3A**, **Tables S1, S2**). The chronological distribution of published documents is presented in **Figure 3B**. Based on the trend line, it was observed that the number of publications on ferroptosis and cancer immunity increased rapidly from 2021 (n = 210, 30.3%) to 2022 (n = 398, 57.3%). As of Feb 10^th^, 2023, 14 documents have been published, and it is likely that more articles will be published in 2023. Among the 530 articles retrieved, 26 types of cancers were mentioned. Among them, lung cancer, hepatocellular carcinoma and breast cancer were the top three most researched cancer types in research associated with ferroptosis and cancer immunity (**Figure 3C**).

### 3.2 Citation and co-citation analysis of the documents

**Figure 4:**
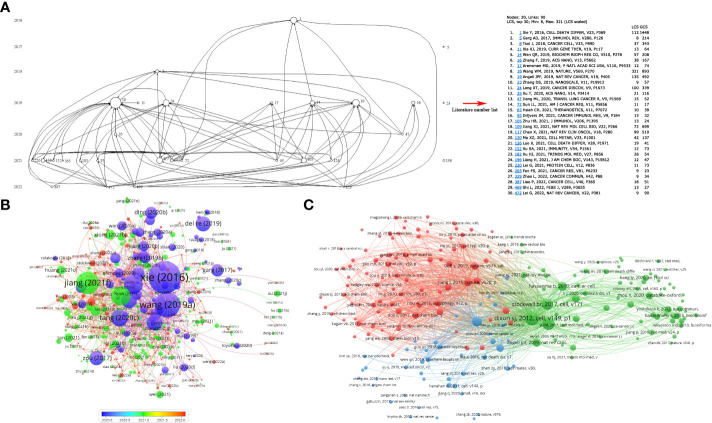
Citation and co-citation of documents. **(A)** the connection of top 30 LCS documents. The analysis was performed by HistCite; **(B)** citation of documents analyzed by VOSviewer; **(C)** co-citation of cited documents analyzed by VOSviewer.

Histcite and VOSviewer were used to analyze the citation and co-citations of 694 documents. The top 10 global citation score (GCS) and local citation score (LCS) documents are shown in **Tables S3, S4**. The GCS was determined by analyzing 694 documents using the Histcite software. The GCS ranged from 1,448 to 281, while LCS ranged from 321 to 37. Wang W.M., 2019” ranked first in LCS among the articles, and “Xie Y., 2016” ranked first in GCS among the reviews. The connection network of citation documents analyzed by Histcite is shown in **Figure 4A**. An article “Wang W.M., 2019” had the highest centrality, indicating that it was the most influential article in the field. Similarly, the connection network visualization through VOSviewer also showed the highest citations of the article “Wang WM, 2019” in article type documents (**Figure 4B**). To analyze the cited references of documents, a co-citation analysis of cited references was performed. As shown in **Figure 4C**, the top 10 most cited articles are listed in **Table 1**. An article by Dixon S.J., 2012 was the most cited publication and was the first article in which ferroptosis was defined.

**Table 1:** Top 10 highly-cited references.

#	Author/Year/Journal	Citation	Percent (%)
1	Dixon SJ, 2012, CELL, V149, P1060, DOI 10.1016/j.cell.2012.03.042	377	54.3
2	Wang WM, 2019, NATURE, V569, P270, DOI 10.1038/s41586-019-1170-y	321	46.3
3	Stockwell BR, 2017, CELL, V171, P273, DOI 10.1016/j.cell.2017.09.021	232	33.4
4	Yang WS, 2014, CELL, V156, P317, DOI 10.1016/j.cell.2013.12.010	196	28.2
5	Hassannia B, 2019, CANCER CELL, V35, P830, DOI 10.1016/j.ccell.2019.04.002	169	24.4
6	Angeli JPF, 2019, NAT REV CANCER, V19, P405, DOI 10.1038/s41568-019-0149-1	135	19.5
7	Liang C, 2019, ADV MATER, V31, DOI 10.1002/adma.201904197	120	17.3
8	Jiang L, 2015, NATURE, V520, P57, DOI 10.1038/nature14344	117	16.9
9	Xie Y, 2016, CELL DEATH DIFFER, V23, P369, DOI 10.1038/cdd.2015.158	112	16.1
10	Bersuker K, 2019, NATURE, V575, P688, DOI 10.1038/s41586-019-1705-2	110	15.9

### 3.3 Association analysis of Keywords

**Figure 5:**
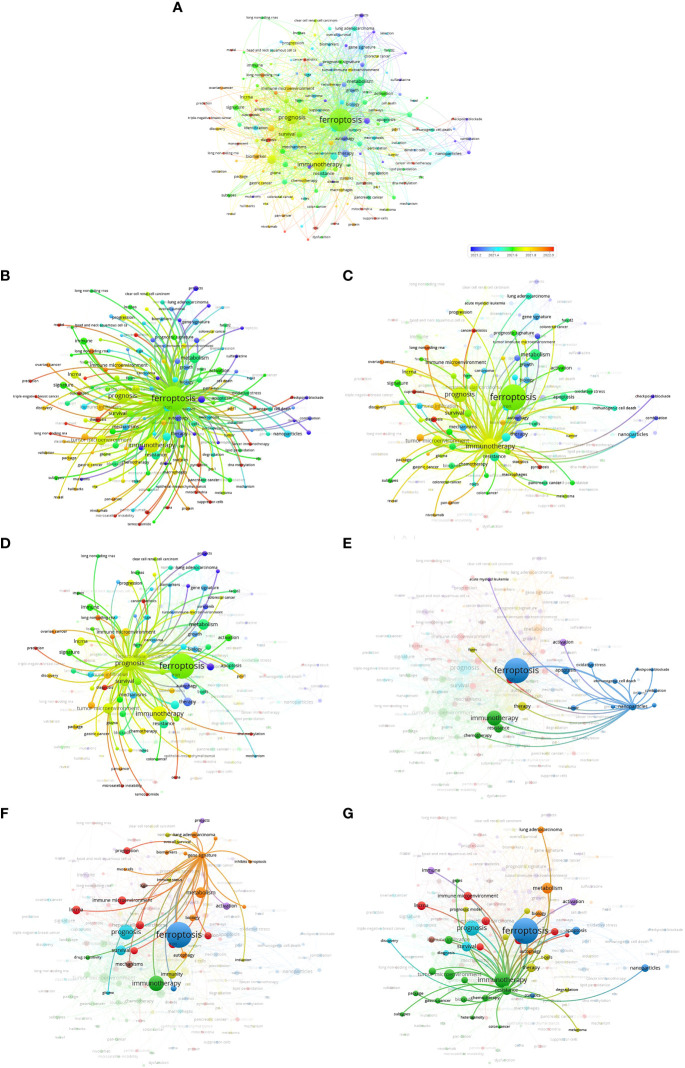
Keyword analysis. **(A–G)**, co-occurrences of keywords by VOSviewer analysis.

During the articles search, 1,585 keywords were retrieved from 530 articles. The minimum number of occurrences of a keyword was 5, and 178 keywords met the threshold. The network visualization map consisted of 173 keywords that were analyzed using VOSviewer. The map shows the co-occurrence relations of keywords by excluding cancer, expression, cells, death, and cell death, which are unrelated to the topic (**Figure 5A**). The size of the circle indicates the occurrence of keywords. As shown in **Figures 5B–D**, the top 3 keywords were ferroptosis, immunotherapy, and prognosis.

Furthermore, some representative keywords were selected based on their close relationship with ferroptosis, immunotherapy, and prognosis, such as resistance, gene signature, and nanoparticles (**Figures 5E–G**). The keyword “resistance” reflects the common problems in clinical treatment. The keyword “gene signature” is a comprehensive biomarker that can predict disease prognosis, and nanoparticles represent a novel way of treating diseases. The above keywords imply that the clinical problem guided the identification of prediction biomarkers and novel treatment options, and the two are the main focus in the field of ferroptosis and cancer immunity.

### 3.4 Author connection analysis

**Figure 6:**
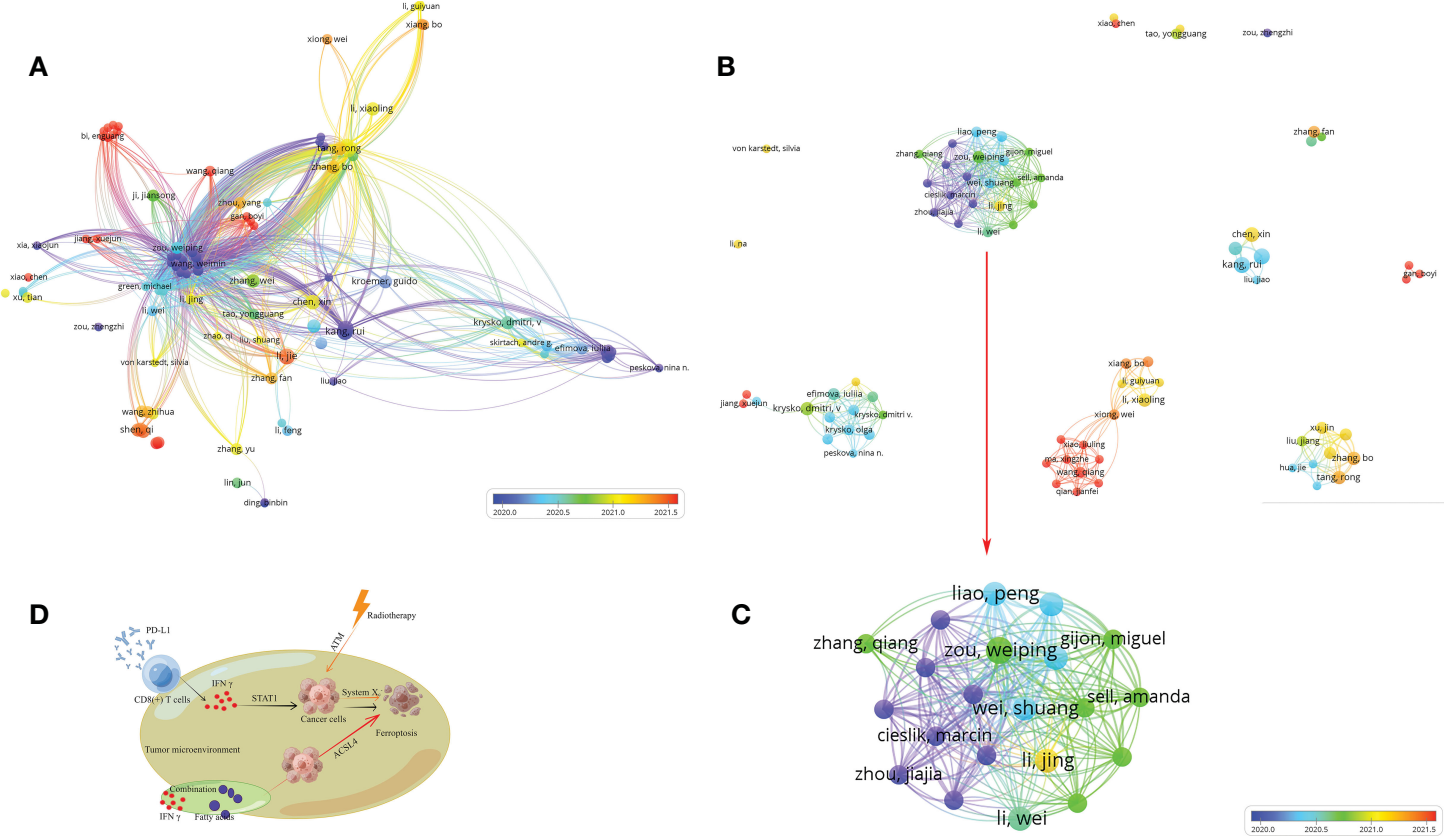
Author and their published articles. **(A)** citation of authors; **(B, C)** co-authorship of authors, **(C)** the red arrow indicates cluster in **(B)**; **(D)** the mechanism of ferroptosis induction and immunotherapy combination. This figure was drawn by Figdraw of HOME for Researchers.

The analysis by Histcite software showed that 4,400 authors contributed to 530 articles. The top 30 authors with the highest total global citation score (TGCS) and total local citation score (TLCS) are shown in **Table 2**. By analyzing the signature of their articles, the 30 authors were correlated with Zou Weiping directly or indirectly. The citation network is shown in **Figure 6A**. Meanwhile, co-authorship analysis was conducted for the 4,400 authors by VOSviewer. A total of 103 authors had published ≥ 2 articles and were cited more than 100 times. The connection network of the above authors is shown in **Figure 6B**. Among them, the largest set of author clusters enriched was Zou Weiping and his colleagues, who headed a group of 21 authors (**Figure 6C**). Furthermore, all articles on ferroptosis and tumor immunity by Zou’s laboratory were retrieved, and four articles have been published so far (**Table S5**). The four articles were read carefully. Among them, one was a review article on IFNγ signaling in tumor immunity in Jan 2022 (21), and the remaining three articles were published in May 2019, Dec 2019, and Apr 2022 respectively. First, they found that IFNγ released by CD8(+) T cells after PD-L1 blockage induced cancer cells ferroptosis via suppressing system Xc- (18). Then, their second article showed that the combination of immunotherapy and radiotherapy also triggered system xc- mediated ferroptosis via IFNγ released by PD-L1 activated CD8(+) T cells and radiotherapy activated Ataxia- Telangiectasia mutated gene (ATM) respectively (19). Based on the above findings, IFNγ and arachidonic acid (AA) were combined further to induce ferroptosis of cancer cells directly. ACSL4, but not system xc-, was discovered to mediate ferroptosis in the third article (20) (**Figure 6D**). Another big cluster of authors comprised Krysko, Dmitri, V, and colleagues, who published three articles, one each in 2019, 2021, and 2022 (**Table S6**, **Figure 6B**). They first discovered that photosens (PS) or photodithazine (PD)-photodynamic therapy (PDT) (PS-PDT or PD-PDT) induced the death of cancer cells. The death could be reversed by ferroptosis inhibitors. The cancer cells are engulfed by bone marrow-derived dendritic cells (BMDC), in which the BMDCs matured and produced IL6 (22, 23). Then they demonstrated that early ferroptotic tumor cells were immunogenic and promoted the phenotypic maturation of BMDCs by acting as vaccines (8).

**Table 2:** Top 30 authors with the highest total global citation score (TGCS) and total local citation score (TLCS).

#	Author	Recs	Percent	TLCS	TLCS/t	TLCSx	TGCS	TGCS/t	TLCR
1	Zou WP	4	0.6	441	94.2	430	1298	279.4	5
2	Kryczek I	3	0.4	439	93.2	428	1283	271.9	4
3	Liao P	3	0.4	439	93.2	428	1283	271.9	4
4	Wang WM	3	0.4	439	93.2	428	1283	271.9	4
5	Wei S	3	0.4	439	93.2	428	1283	271.9	4
6	Choi JE	2	0.3	421	84.2	410	1232	246.4	2
7	Cieslik M	2	0.3	421	84.2	410	1232	246.4	2
8	Georgiou G	2	0.3	421	84.2	410	1232	246.4	2
9	Lang XT	2	0.3	421	84.2	410	1232	246.4	2
10	Lawrence TS	2	0.3	421	84.2	410	1232	246.4	2
11	Szeliga W	2	0.3	421	84.2	410	1232	246.4	2
12	Vatan L	2	0.3	421	84.2	410	1232	246.4	2
13	Zhou JJ	2	0.3	421	84.2	410	1232	246.4	2
14	Gijon M	2	0.3	339	73.2	329	944	204.1	3
15	Johnson JK	2	0.3	339	73.2	329	944	204.1	3
16	Kennedy PD	2	0.3	339	73.2	329	944	204.1	3
17	Sell A	2	0.3	339	73.2	329	944	204.1	3
18	Green M	2	0.3	323	65.2	313	908	186.1	2
19	Li J	15	2.2	323	65.2	313	1123	248.6	20
20	Chan TA	1	0.1	321	64.2	311	893	178.6	1
21	Chinnaiyan A	1	0.1	321	64.2	311	893	178.6	1
22	Gu W	1	0.1	321	64.2	311	893	178.6	1
23	Lamb C	1	0.1	321	64.2	311	893	178.6	1
24	Li GP	1	0.1	321	64.2	311	893	178.6	1
25	Li W	3	0.4	321	64.2	311	944	195.6	2
26	Liu R	2	0.3	321	64.2	311	893	178.6	1
27	Stone E	1	0.1	321	64.2	311	893	178.6	1
28	Tanno Y	1	0.1	321	64.2	311	893	178.6	1
29	Xia HJ	1	0.1	321	64.2	311	893	178.6	1
30	Zhang HJ	2	0.3	321	64.2	311	894	179.1	2

TLCS, Total Local citation score; TLCS/t, Total local citation scores per year; TGCS, Total global citation score; TGCS/t, Total global citation scores per year; TLCSx, Total Local citation score excluding self-citations; TLCR, Total local cited references.

### 3.5 Nanoparticle therapy-related articles

**Figure 7:**
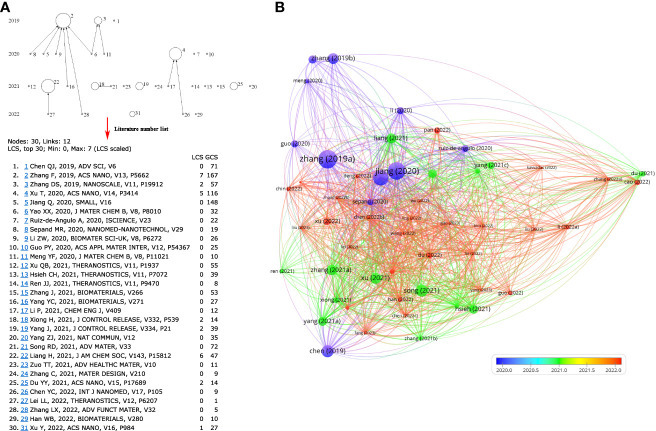
Data mining in nanoparticle-related articles. **(A)** Top 30 LCS of 53 documents. The analysis was performed by HistCite, the note of Top 30 LCS literature; **(B)** Literature coupling analysis by VOSviewer.

According to the connection between ferroptosis and anti-tumor immunity, a series of nanomedicine articles have emerged. The 530 articles were refined by tapping the keyword “nanoparticles”, and 53 documents were collected (**Table S7**). Next, the source of 53 articles was investigated, and there were 28 journals in total (**Table 3**). The top four publications were occupied by BIOMATERIALS (6 records), ACS NANO (4 records), SMALL (4 records), and THERANOSTICS (4 records). The top ten most cited articles are listed in **Table 4**. Furthermore, the top 30 LCS were analyzed by Histcite software. Article by Zhang F, Li F, Lu GH, Nie WD, Zhang LJ, et al. Engineering Magnetosomes for Ferroptosis/Immunomodulation Synergism in Cancer. ACS NANO. 2019 MAY; 13 (5): 5662-5673” had the best center direction and was the most cited (**Figure 7A**). This article was the first nanomedicine report on ferroptosis and immunotherapy. The article was published in May 2019, almost simultaneously with an influential article by Wang W.M. Wang W.M. (2019) was submitted on Apr 2018 from the USA, and Zhang F. (2019) was submitted on Jan 2019 from China. A literature coupling analysis was conducted, and the total link strength of the 53 articles was ranked from 156 to 23. The top three most linked articles were Du (2022), Jiang (2020), and Sepand (2020) (**Figure 7B**). 51 articles (excluding one hypothesis and one review from 53 records) on nanoparticles are selected to summarize the details of nanoparticles in fighting cancer. The statistics about the name, composition, and function of the nanoparticles are listed in **Table 5**. Most of the nanoparticles mentioned above were constructed based on the synergistic effect of ferroptosis and immunotherapy.

**Table 3:** Documents on nanoparticles involved in ferroptosis and cancer immunity.

#	Journal	Publication records	Percent	TLCS	TLCS/t	TGCS	TGCS/t	TLCR
1	BIOMATERIALS	6	11.3	0	0	109	41.17	3
2	ACS NANO	4	7.5	15	3.82	324	80.57	0
3	SMALL	4	7.5	0	0	165	45.5	5
4	THERANOSTICS	4	7.5	0	0	103	34.5	1
5	CHEMICAL ENGINEERING JOURNAL	3	5.7	0	0	14	5	2
6	JOURNAL OF CONTROLLED RELEASE	3	5.7	4	1.33	53	17.67	1
7	JOURNAL OF NANOBIOTECHNOLOGY	3	5.7	0	0	12	6	1
8	ACS APPLIED MATERIALS & INTERFACES	2	3.8	0	0	32	9.75	1
9	ADVANCED FUNCTIONAL MATERIALS	2	3.8	0	0	22	11	3
10	ADVANCED HEALTHCARE MATERIALS	2	3.8	0	0	11	3.67	2
11	ADVANCED SCIENCE	2	3.8	0	0	78	17.7	0
12	JOURNAL OF MATERIALS CHEMISTRY B	2	3.8	0	0	42	10.5	3
13	ACTA BIOMATERIALIA	1	1.9	0	0	10	5	1
14	ADVANCED MATERIALS	1	1.9	0	0	72	24	1
15	BIOMATERIALS SCIENCE	1	1.9	0	0	26	6.5	1
16	INTERNATIONAL JOURNAL OF NANOMEDICINE	1	1.9	0	0	9	4.5	1
17	ISCIENCE	1	1.9	0	0	22	5.5	0
18	JOURNAL FOR IMMUNOTHERAPY OF CANCER	1	1.9	0	0	11	5.5	0
19	JOURNAL OF COLLOID AND INTERFACE SCIENCE	1	1.9	0	0	13	6.5	0
20	JOURNAL OF DRUG TARGETING	1	1.9	0	0	0	0	0
21	JOURNAL OF THE AMERICAN CHEMICAL SOCIETY	1	1.9	6	2	47	15.67	0
22	MATERIALS & DESIGN	1	1.9	0	0	9	3	0
23	MATERIALS TODAY CHEMISTRY	1	1.9	0	0	0	0	0
24	MEDICAL HYPOTHESES	1	1.9	0	0	0	0	0
25	NANOMEDICINE-NANOTECHNOLOGY BIOLOGY AND MEDICINE	1	1.9	0	0	19	4.75	1
26	NANOSCALE	1	1.9	2	0.4	57	11.4	0
27	NATIONAL SCIENCE REVIEW	1	1.9	0	0	0	0	0
28	NATURE COMMUNICATIONS	1	1.9	0	0	35	11.67	0

TLCS, Total Local citation score; TLCS/t, Total local citation scores per year; TGCS, Total global citation score; TGCS/t, Total global citation scores per year; TLCSx, Total Local citation score excluding self-citations; TLCR, Total local cited references.

**Table 4:** The top 10 most cited articles in the field of nanoparticles.

#	Date/Author/Journal	LCS	LCS/t	LCSx	GCS	GCS/t	NA	LCR	CR
1	Zhang F, Li F, Lu GH, Nie WD, Zhang LJ, et al. Engineering Magnetosomes for Ferroptosis/Immunomodulation Synergism in Cancer ACS NANO. 2019 MAY; 13 (5): 5662-5673	7	1.4	7	167	33.4	11	0	37
2	Jiang Q, Wang K, Zhang XY, Ouyang BS, Liu HX, et al. Platelet Membrane-Camouflaged Magnetic Nanoparticles for Ferroptosis-Enhanced Cancer Immunotherapy SMALL. 2020 JUN; 16 (22): Art. No. 2001704	0	0	0	148	37	7	1	66
3	Xu T, Ma YY, Yuan QL, Hu HX, Hu XK, et al. Enhanced Ferroptosis by Oxygen-Boosted Phototherapy Based on a 2-in-1 Nanoplatform of Ferrous Hemoglobin for Tumor Synergistic Therapy ACS NANO. 2020 MAR 24; 14 (3): 3414-3425	5	1.25	4	116	29	9	0	54
4	Song RD, Li TL, Ye JY, Sun F, Hou B, et al. Acidity-Activatable Dynamic Nanoparticles Boosting Ferroptotic Cell Death for Immunotherapy of Cancer ADVANCED MATERIALS. 2021 AUG; 33 (31): Art. No. 2101155	0	0	0	72	24	11	1	38
5	Chen QJ, Liu LS, Lu YF, Chen XL, Zhang YJ, et al. Tumor Microenvironment-Triggered Aggregated Magnetic Nanoparticles for Reinforced Image-Guided Immunogenic Chemotherapy ADVANCED SCIENCE. 2019 MAR 20; 6 (6): Art. No. 1802134	0	0	0	71	14.2	13	0	43
6	Zhang DS, Cui P, Dai ZC, Yang BC, Yao XX, et al. Tumor microenvironment responsive FePt/MoS2 nanocomposites with chemotherapy and photothermal therapy for enhancing cancer immunotherapy NANOSCALE. 2019 NOV 14; 11 (42): 19912-19922	2	0.4	0	57	11.4	8	0	44
7	Xu QB, Zhan GT, Zhang ZL, Yong TY, Yang XL, et al. Manganese porphyrin-based metal-organic framework for synergistic sonodynamic therapy and ferroptosis in hypoxic tumors THERANOSTICS. 2021; 11 (4): 1937-1952	0	0	0	55	18.33	6	0	45
8	Zhang J, Yang J, Zuo TT, Ma SY, Xokrat N, et al. Heparanase-driven sequential released nanoparticles for ferroptosis and tumor microenvironment modulations synergism in breast cancer therapy BIOMATERIALS. 2021 JAN; 266: Art. No. 120429	0	0	0	53	17.67	10	0	68
9	Liang H, Wu XY, Zhao GZ, Feng K, Ni KY, et al. Renal Clearable Ultrasmall Single-Crystal Fe Nanoparticles for Highly Selective and Effective Ferroptosis Therapy and Immunotherapy JOURNAL OF THE AMERICAN CHEMICAL SOCIETY. 2021 SEP 29; 143 (38): 15812-15823	6	2	6	47	15.67	6	0	46
10	Hsieh CH, Hsieh HC, Shih FS, Wang PW, Yang LX, et al. An innovative NRF2 nano-modulator induces lung cancer ferroptosis and elicits an immunostimulatory tumor microenvironment THERANOSTICS. 2021; 11 (14): 7072-7091	0	0	0	39	13	7	0	63

LCS, Local citation score; LCS/t, Local citation scores per year; GCS, Global citation score; GCS/t, Global citation scores per year; LCSx, Local citation score excluding self-citations; LCR, Local cited references; NA, Number of authors; CR, Cited references.

**Table 5:** Nanoparticles name, composition, and function of the 51 articles.

Nanoparticle name	Components	Function	Refs
Nanoformulation (CP)	Ferroptosis-inducing cannabinoid nanoparticles and immunostimulatory Poly(I:C)	Activating ferroptosis-immunotherapy pathways	(24)
A novel biomimetic nanoplatform	Photosensitizer chlorin e6 (Ce6), hemin and PEP20 (CD47 inhibitory peptide)	Integrating oxygenboosted PDT, ferroptosis activation and CD47-SIRP alpha blockade	(25)
Fe/PEI-Tn	Polyethyleneimine (PEI), Fe3+ and the modification of bifunctional peptides Tn	Blocking the PD-1/PD-L1 pathway, activating macrophages, reversing the phenotype of pro-tumour M2-type macrophages	(26)
Zero-valent-iron nanoparticle (ZVI-NP)	ZVI@Ag, ferrous sulfate (FeSO4) and trisodium citrate (Na3C6H5O7) dehydrate	Shifting pro-tumor M2 macrophages to anti-tumor M1, decreasing the population of regulatory T cells, downregulating PD-1 and CTLA4 in CD8(+) T cells	(27)
mFe(SS)/DG	Cancer cell membrane coated metal organic framework (MOF), glucose oxidase (GOx) and doxorubicin (DOX)	Releasing tumor antigens to initiate antitumor immunity	(28)
HLCaP nanoreactors (NRs)	CaCO3-assisted double emulsion, lipoxidase, hemin and poly(lactic-co-glycolic acid) (PLGA)	Producing cytotoxic lipid radicals and priming antitumor immunity	(29)
FPBC@SN	ferritin, pH-sensitive molecular-switch,sorafenib (SRF) and IDO inhibitor (NLG919)	Promoting ferroptosis and arouse tumor immunity	(30)
CCM@Mn@MSN-Pt(IV), CMnMPt	Mn ions-doped mesoporous silica nanoparticles (Mn@MSN), cisplatin prodrug (Pt(IV)) and cancer cell membrane cloaking	Inducing ferroptosis-mediated ICD and recruiting cytotoxic T lymphocytes cells	(31)
m@Au-D/B NCs	Cancer cell membrane-camouflaged gold nanocage, doxorubicin (DOX) and L-buthionine sulfoximine (BSO)	Inducing ferroptosis and repolarizing the tumor-associated macrophages (TAMs) from protumor (M2) phenotype to anti-tumor (M1) phenotype	(32)
NLC/H(D + F + S) NPs	Heparanase (HPSE)-driven sequential released nanoparticles, beta-cyclodextrin (beta-CD) grafted heparin (NLC/H(D + F + S) NPs), doxorubicin (DOX), ferrocene (Fc), and TGF-beta receptor inhibitor (SB431542)	Modulating tumor microenvironment and activating ferroptosis pathway	(33)
Ferumoxytol	Iron oxide nanoparticles	Ferumoxytol mediated ferroptosis and increased NK cells’ cytotoxic function	(34)
C-RAuNC	Cancer cell membrane coated gold nanocages and RSL3	Synergistic treatment of ferroptosis and photothermal therapy (PTT) initiate effective anti-tumor immunity	(35)
A nanozyme-based formulation	Ultrasmall CaO2, Fe3O4 nanoparticles (NPs), dendritic mesoporous silica nanoparticles (DMSN) and the pH-responsive membrane	Synergized efficient ferroptosis with immunomodulation	(36)
FePt/BP-PEI-FA NCs	FePt nanoparticles (FePt NPs) and ultrathin black phosphorus nanosheets (BPNs)	Synergistic effect of photothermal therapy (PTT), photodynamic therapy (PDT), and chemodynamic therapy (CDT)	(37)
zinc-fluorouracil metallodrug networks (Zn-Fu MNs)	Zn and Fu	Enhanced ROS production and immune activation	(38)
Biomimetic magnetic nanoparticles, Fe3O4-SAS @ PLT	Sulfasalazine (SAS), mesoporous magnetic nanoparticles (Fe3O4) and platelet (PLT) membrane camouflage	Sensitize effective ferroptosis and produce mild immunogenicity	(39)
A Hypoxia-responsive nanoelicitor (HRNE)	Immune-elicitable polyphenols, Chlorogenic acid (CA), Mitoxantrone (MIT), Fe3+ ions and hypoxia-responsive hybrid liposomal membrane	Fenton reaction and activated tumoricidal immunity	(40)
AuNRs&IONs@Gel	A gel delivery platform, embedded gold nanorods (AuNRs), and iron oxide nanoparticles (IONs)	Induce ferroptosis and transfer M2-like phenotype into the antitumor M1-like phenotype	(41)
A biomimetic magnetosome	Fe304 magnetic nanocluster (NC), pre-engineered leukocyte membranes as the cloak, TGF-fl inhibitor (Ti), and PD-1 antibody (Pa)	Ferroptosis/immunomodulation synergism in cancer	(42)
GCMNPs	A leukocyte membrane coated poly (lactic-co-glycolic acid) and glycyrrhetinic acid	Combination of GCMNPs, ferumoxytol and anti-PD-Ll improve T-cell immune response synergistically	(43)
IrFc1	A ferrocene-containing Ir(III) photosensitizer	Causing ferroptosis and promoting immunogenic cell death (ICD)	(44)
ETP-PtFeNP	Enolase targeting peptide, Pt-prodrug and Fe3O4 nanoparticles	ICD-associated antitumor immune responses	(45)
ZnP@DHA/Pyro-Fe particles	Cholesterol derivative of DHA (Chol-DHA) and Pyropheophorbide-iron (PyroFe)	Sensitizing non-immunogenic cancers to anti-PD-L1 immunotherapy	(46)
IONVs	Iron oxide-loaded nanovaccines	Improving immunostimulatory capacity and targeting tumor cell ferroptosis	(47)
FeCO-IR820@FeIIITA	Thermosensitive boronic acid group -containing CO prodrug, tannic acid (TA), iron (Fe) and near-infrared (NIR) photothermal agent IR820	Enhancing ferroptosis and CTLA-4 blockade immunotherapy	(48)
siProminin2	The iron oxide nanoparticles, polymers and oxaliplatin attached	Inhibiting the secretion of tumor cell-derived exosomes and enhancing the immune activation	(49)
Acidity-Activatable Dynamic Nanoparticles	Ionizable block copolymer, acid-liable phenylboronate ester (PBE) dynamic covalent bonds and a glutathione peroxidase 4 inhibitor RSL-3.	Recruiting tumor-infiltrating T lymphocytes for IFNγ and sensitizing ferroptosis	(50)
AuPB@LMHep	Fusing hepcidin, leukemia cell-membrane vesicles, gold nanoparticles (AuNPs) and hollow mesoporous Prussian Blue	AuNPs triggered ferroptosis and AuPB@LMHep enhanced cytotoxic tumor-infiltrating T cells effect	(51)
TPA-NDTA NP	High-performance photothermal nanoparticle	Promoting ferroptosis and evoking ICD through ferroptosis pathway	(52)
CM CTNPs@OVA	Solid mes-oporous copper telluride nanoparticles, ovalbumin (OVA), mesoporous, and melanoma cell membrane	Initiating ferroptosis and ICD by DC maturation and T cells recruitment	(53)
Fe3O4@Chl/Fe CNPs	Cluster-structured nanoparticles (CNPs), Fe3O4 and iron chlorophyll (Chl/Fe) photosensitizers	Reprogramming of the tumor microenvironment	(54)
A “closed-loop” therapy	Copper silicate, iron silicate mesoporous hollow Nanospheres, Au nanoparticles and an immune adjuvant resiquimod R848	Enhancing ferroptosis and immunogenic cell death (ICD)	(55)
iRGD-bccUSINPs	iRGD peptide, Fe core around 2 nm and an iron oxide shell less than 0.7 nm	Inducing ferroptosis and immunogenetic cell death	(56)
Mn-MOF	A manganese porphyrin-based metal-organic framework	Enhancing SDT (ultrasound (US)-triggered sonodynamic therapy) and ferroptosis	(57)
Mitochondrial-targeting liposomal nanoparticles (abbreviated MLipRIR NPs)	The encapsulation of R162 (inhibitor of glutamate dehydrogenase 1 [GDH1]), IR780 (a hydrophobic sonosensitizer) and the lipid bilayer	Causing severe ferroptosis and triggering immunogenic cell death (ICD)	(58)
Bi2Te3-Au/Pd	near-infrared (NIR-II) photothermal-nanocatalyst, Bi2Te3 nanosheets and ultrasmall Au/Pd bimetallic nanoparticles	Modulating the TME and enhancing ferroptosis	(59)
Lp-IO	Liposomes, PEG-coated 3 nm gamma-Fe2O3 nanoparticles and bilayer	Initiating ferroptosis in cancer cells	(60)
GNRa-CSP12	Gold nanorods (GNRs), a binary surfactant mixture of hexadecyltrimethylammonium bromide and sodium oleate	Abrogating endogenous Fe2+-dependent m(6)A demethylase activity	(61)
DOX-TAF@FN	Doxorubicin (DOX), tannic acid (TA)-iron (Fe) networks (for short, TAF) and fibronectin (FN)	Inducing immunogenic cell death through enhanced ferroptosis of cancer cells	(62)
PFTT@CM	Polyvinyl pyrrolidone (PVP) dispersed nanoscale metal-organic framework (NMOF) of Fe-TCPP, hypoxia-activable prodrug tirapazamine (TPZ) and the cancer cell membrane (CM)	Triggering ferroptosis and enhancing photodynamic therapy (PDT) efficacy	(63)
CCR2(+)-Fe-M1-Nys	M1 macrophages, up-regulated CCR2 expression, as Fe3O4 nanoparticles carrier, exosome-mimic nanovesicles (denoted as CCR2(+)-Fe-M1-Nys)	Facilitating ferroptosis and inducing macrophages repolarization	(64)
Monodispersed ferrihydrite nanoparticles	Monodispersed ferrihydrite nanoparticles	Apoptosis- and ferroptosis of cancer cell and tumor associated macrophage (TAM) polarization from the tumor-promoting M2 type to the tumor-killing M1 type	(65)
Multifunctional FePt/MoS2-FA nanocomposites (FPMF NCs)	Anchoring FePt nanoparticles, folic acid (FA) and MoS2 nanosheets	Cytosine-guanine (CpG ODNs) combined with systemic checkpoint blockade therapy using an anti-CTLA4 antibody	(66)
SRF@Hb-Ce6	Hemoglobin (Hb), the photosensitizer chlorin e6 (Ce6), a 2-in-1 nanoplatform (SRF@Hb-Ce6) and Sorafenib (SRF, ferroptosis promotor)	Recruiting immune cells to secrete IFN-gamma and sensitizing Fe-dependent ferroptosis	(67)
multifunctional nanotherapeutic agent FePt@COP-FA nanocomposites (FPCF NCs)	Magnetic FePt-cubes, carboxylated by 3-(4-hydroxyphenyl) propionic acid (DHCA), benzidine (BD), 1,3,5-triformylphloroglucinol (Tp), COP shells and HS-PEG-FA	Activating apoptosis, ferroptosis and specific immune response	(68)
GBM-targeted drug delivery system (Fe3O4-siPD-L1@M–(Bv2))	BV2 membrane, -S-S-, siPD-L1-SH, Fe3O4-SH	Inducing ferroptosis of GBM cells and maturation of DC cell	(69)
MP@PI	The metal-organic framework (MOF), polydopamine (PDA), IR820 and piperlongumine (PL)	Eliciting ferroptosis and pyroptosis	(70)
GNPIPP12MA	FTO inhibitor and GSH-bioimprinted nanocomposites	GNPIPP12MA selectively targeting leukemia blasts and inducing ferroptosis	(71)
RSL3@O2-ICG NBs	Nanobubbles (NBs), sonosensitizer Indocyanine green (ICG) and RAS-selective lethal (RSL3, ferroptosis promoter)	Enhancing SDT and ferroptosis	(72)
A nano-activator (DAR)	Doxorubicin (DOX), tannic-acid (TA) and IR820	Facilitating ferroptosis and immunotherapy respectively.	(73)
MiR-21-3p-loaded gold nanoparticles	MiR-21-3p and gold nanoparticles	Activating IFN-gamma-mediated ferroptosis	(74)

### 3.6 Gene signature-related articles

**Figure 8:**
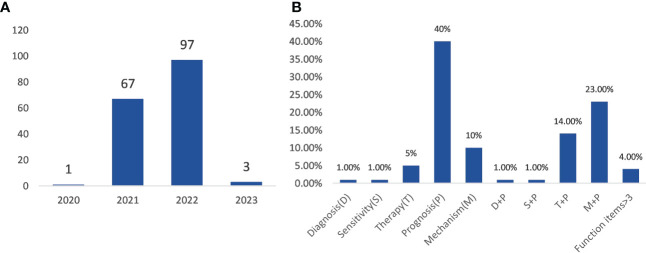
Analysis of gene signature-related articles. **(A)** the time distribution of gene signature-related article publications; **(B)** the ratio of research direction to gene signature.

A total of 168 articles out of 530 articles contained the keywords “gene signature” (**Table S8**). The publication period ranged from 2020 to 2023. As shown in **Figure 8A**, only one article was published in 2020, 67 articles in 2021, and 97 articles in 2022, three articles in 2023. These articles were screened by reading the title and abstract, and the articles were divided into 10 clusters according to the directions of a gene signature in ferroptosis and cancer immunity (**Figure 8B**). These articles mainly focused on five aspects of gene signature in the field of ferroptosis and cancer immunity, including diagnosis, sensitivity, therapy, prognosis, and mechanism. The number of articles on the above directions either alone or in combinations, is shown in **Figure 8B**. Most of these gene signature-related articles were focused on the direction of prognosis, including single and combined directions.

## 4 Discussion

Since its first discovery, ferroptosis has become popular worldwide. In the last ten years, there has been a sharp increase in the number of documents on ferroptosis and tumor immunity, and these two interrelated fields have gained significant attention in the recent three years (**Figure 3B**). The increase in the number of articles on cancer and ferroptosis was still observed until the end of 2022. Among these publications, the documents on lung cancer are the most published, maybe because lung cancer has high morbidity and attracts more attention from researchers. Overall, our findings imply that more researchers might focus on ferroptosis and cancer immunity. Keywords analysis in this study presents the hotspots in the field of ferroptosis and cancer immunity, such as prognosis, gene signature, and nanoparticles, which have the potential for prediction and therapy strategy. Gene signature for drug sensitivity or prognosis prediction is popular for big data application in cancer research development based on real data analysis and model validation (75, 76). Overall, the attention on drug sensitivity and treatment outcomes of ferroptosis and cancer immune has increased. In the next few years, more articles on ferroptosis and cancer immunity are anticipated to be published.

Dixon (2012) and Wang (2019) were the most co-cited references. Wang W.M. et al. reported that IFNγ released by CD8(+) T cells after PD-L1 blockage triggered ferroptosis mediated by system xc-, indicating the connection of immunotherapy and ferroptosis and the influence of tumor microenvironment. Wang’s colleagues (Zou’s lab) focused on the synergistic effect of immunotherapy and ferroptosis. Zou’s group contributed a lot to the synergy theory of ferroptosis and immunotherapy. Screening of their articles published in recent years revealed that they mainly focused on cancer immunotherapy. Apart from ferroptosis, they also researched on autophagy and how it may work in synergy with immunotherapy to treat cancer (77). Moreover, T cells and macrophages played a significant role in cancer immunotherapy, which was evident in their published papers (78). From 2019 to 2022, they published more than 20 articles on the mechanism of immunotherapy and some combination treatments, suggesting their persistent efforts in cancer research (**Table S9**).

In clinical, only a small percentage of patients responded to immune checkpoint inhibitors (ICIs) such as PD-L1. Resistance to checkpoint inhibitors was significant (79). Coincidently, immunotherapy and ferroptosis work in synergy to overcome the resistance of single cancer treatment especially immunotherapy. Apart from ferroptosis, necroptosis and apoptosis also act as important role in killing cancer, However, Krysko, Dmitri et al. showed that ferroptosis had unique immunogenic characteristics. In his research, early ferroptotic tumor cell death stimulated tumor immunity and enhanced immune memory (8). Ferroptotic cancer cells released three DAMPs, including HMGB1, ATP, and CRT, promoting immunogenicity that aided in overcoming resistance to the cancer drug. Furthermore, various lipids released by ferroptotic cancer cells were shown to target immune cells such as DCs and CD8+ T cells to exert the anti-tumor immunity effects (8, 80).

**Figure 9:**
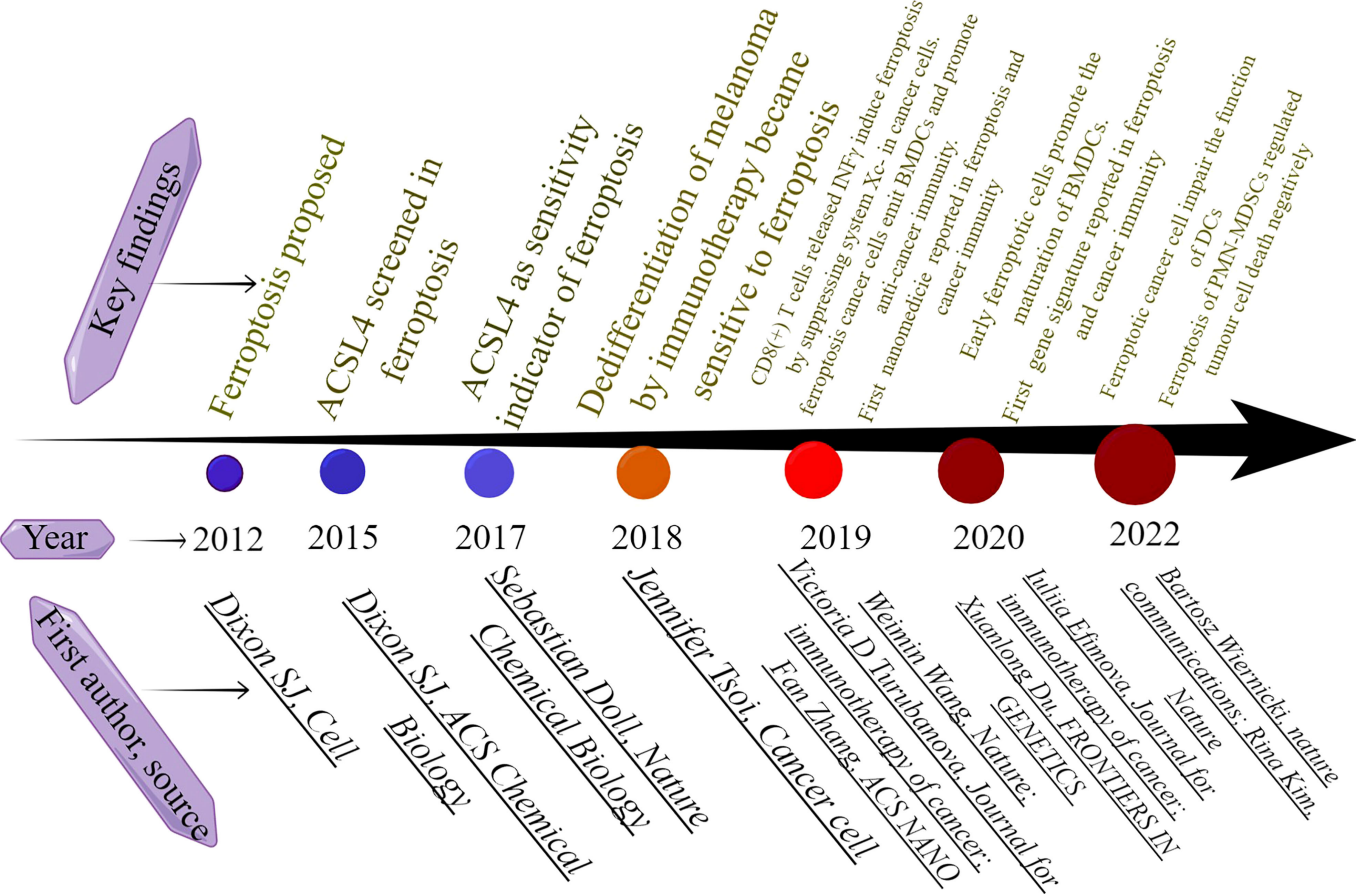
The milestones in ferroptosis and cancer immunity. This figure was drawn by Figdraw of HOME for Researchers.

1183405However, conflicting opinions still exist. Research by Peter Vandenabeele‘s group showed that ferroptotic cancer cells impair the function of DCs and cannot stimulate the immune response in cancer patients (81). In addition, another research by this group also showed that ferroptosis induction impairs the recruitment of immune cells and may act as a biomarker to predict the poor outcome of cancer patients (82). In cancer immunity, T lymphocytes are the major regulators of anti-cancer immunity. Antigen-presenting cells (APCs) including dendritic cells (DCs) and macrophages present antigenic peptides of cancer cells to T cells, the peptide binding major histocompatibility complex molecules (MHCs) is exposed on the cell surface to be recognized by T cell receptors (TCRs) and form TCR-peptide-MHC complex that activates the T cell (83). Therefore, the activation of APC cells and T cells plays a major role in anti-cancer immunity. Initially, the cytokine IFNγ was identified to be primarily secreted by CD8+ T cells and known to affect the dedifferentiation degree of melanomas cells. The dedifferentiation subtype of melanomas cells is promoted by IFNγ in response to ferroptosis inducers. It is therefore speculated that ferroptosis inducers in combination with cytokine stimulators may promote anti-tumor immunity (17). Subsequently, a research published on the nature journal demonstrated that the IFNγ secretion of CD8+ T cells activated by PD-L1 blockage promoted ferroptosis of CD45^-^ ID8 cells, and combination of cyst(e)inase and PD-L1 blockage significantly increased the anti-cancer efficacy (18). Subsequently, Iuliia Efimova and her colleagues suggested that when early ferroptotic cancer cells (specifically MCA205 cells) are taken up by BMDCs, they can trigger the activation of BMDCs by inducing the expression of CD80, CD86, and MHCII on BMDCs, potentially serving as a cancer treatment vaccine. They confirmed the strong immunogenic potential of early ferroptotic cancer cells (8). Nevertheless, conflicting findings emerged from Peter Vandenabeele’s laboratory, wherein they replicated the experimental conditions used by Iuliia Efimova’s team but discovered that early ferroptotic cancer cells are non-immunogenic and do not trigger anti-tumor immunity. Moreover, they discovered that the corpses of ferroptotic cancer cells were negatively correlated with the activation and proliferation of CD8+ T cells (81). A recent study revealed that pathologically activated neutrophils and myeloid-derived suppressor cells (PMN-MDSCs) in human tumors, exhibiting elevated ferroptosis levels, are particularly susceptible to ferroptosis via the regulation of FATP2. Consequently, the functionality and proliferation of T cells within the tumor microenvironment are impeded, with the sensitivity of ferroptosis in PMNs being contingent on hypoxia. Notably, the combination of ferroptosis inhibition and PD-1 antibody has been found to augment antitumor immunity (84). The cells used in the study were obtained from tumor-bearing animals not directly from cell lines, and the hypoxia condition of tumor microrenvironment (TME) was developed *in vitro*. Thus, the results of the study may more accurately match clinical findings. An alternative hypothetical explanation for the conflicting viewpoints is the immune cell status in response to ferroptotic signals. Specifically, if T cells or DC cells are already activated by immunotherapy within the tumor microenvironment, then the induction of ferroptosis may potentially synergize with the anti-tumor immunity. Conversely, if the antitumor immunity is unresponsive, the hierarchy of cell death within the tumor microenvironment is determined by the varying sensitivity of cell types to ferroptosis. Under such circumstances, if immune cells are more susceptible to ferroptosis, then Rina Kim’s findings may be more comprehensible. Further evidence from scholars is necessary to validate the veracity of these findings. Overall, research into the intersection of ferroptosis and tumor immunity has made significant progress in recent years, as evidenced by the milestones highlighted in **Figure 9**.

With the development of the synergy theory of ferroptosis and cancer immunity, a series of applications that show great potential, such as nano-medicine and gene signature-based prediction, have emerged. Nanoparticles are applied in combination with ferroptosis, and immunotherapies. In three years, 51 articles about nanoparticle designs based on ferroptosis and immunotherapies targets had already been published. This number of publications indicated that researchers are confident in potentially inducing synergistic effects on ferroptosis and immunotherapies. The design of nanoparticles for inducing ferroptosis primarily focuses on targeting ROS, iron overload, GSH, and certain ferroptosis-regulated molecules, including system xc-, GPX4, and ACSL4 (85–90). ROS is one of the most important mechanisms that is applied during the formulation of nano-medicines. On one hand, physiological ROS accumulation can promote the death of cancer cells. Therefore, the nanoparticles such as PPS designed by Xianwen Wang and his colleagues aimed to increase the accumulation of H_2_O_2_, decrease the amounts of glutathione (GSH) and cause death by targeting cancer cells (91). As such, PPS may serve as an ideal complement to ferroptosis inducers and/or immunotherapies, thereby improving the effectiveness of cancer treatment. However, the rapid buildup of ROS in cancer cells over a short period can result in a severe inflammatory response. To resolve the excess ROS problems caused by PPT, Xianwen Wang and his colleagues designed ultrasmall ZrC NDs, which eliminates ROS to decrease inflammation caused by PTT treatment for glioma (92). Subsequently, ultrasmall ZrC–PVP nanodots (NDs) (ZrC–PVP NDs) based on the combination of PTT–RT photothermal therapy (PTT) -Radiation therapy (RT) was designed for glioma treatment (93). Furthermore, they attempted to disrupt the respiration process and modify the oxidation state of cancer cells by continuously generating H_2_ through the transfer of MgG rods into the cancer cells. This approach has the potential to trigger a robust synergistic effect with ferroptosis inducers and immunotherapies (94). As an increasing number of nano-medicines for cancer treatment based on ROS, GSH, and iron are being explored, the long-term accumulation and biodegradability of these medicines may prove critical to their clinical translation (95). So far, nanoparticles combining ferroptosis induction and immunotherapies are very diverse. However, there is still a long way to go for patients to benefit, considering the transformation, clinic trial, production volume, and economy.

Apart from nanoparticles, the gene signatures for prognosis prediction in ferroptosis and cancer immunotherapies are hotspots that attract the attention of so many researchers. The traction on gene signature indicates that it may be promising for evaluating patients in the future. The gene signature research is based on big data and data mining, which reflects the principles of gene presentation and paves the way for treatment based on translation research. Apart from prediction, prompt treatment based on the combination of ferroptosis induction and immunotherapy is urgent, but currently, the strategy is full of challenges.

In summary, publications on ferroptosis and tumor immunotherapy have increased rapidly in the last 3 years, and most studies were on lung cancer. Zou Weiping’s laboratory contributed a lot and published the most influential articles. The mechanism, prediction, and therapy directions of this topic are hotspots in the research trends. Particularly, the development of nanomedicines based on the benefit of combining ferroptosis and immunotherapy has been rapid, and it presents promising hotspots in future research.

### 4.1 Limitation

Nevertheless, this bibliometric study has limitations that should be mentioned. The search date in this study in the Web of Science Core Collection was Feb 10^th^, 2023. Since the data is constantly updated, some parts of documents in this field, from the search date to the publication date, are not included. Another limitation is that the keywords tapped are TS and are mostly present in the title or the abstract. Therefore, some useful keywords in the main text could be missed. The third reason is that the database selected for the study may not be comprehensive enough to cover all documents in this field. Last but not least, there are conflicting views on whether ferroptosis is immunogenic or not and its role in affecting immune cells. No other group provides external evidence to support or disprove their hypothesis. Maybe it will be revealed by more research in the near future. Other limitations in this study have been previously described (96, 97).

## Data availability statement

The original contributions presented in the study are included in the article/**Supplementary Material**. Further inquiries can be directed to the corresponding author.

## Author contributions

ZW and CY designed the search strategy and prepared the manuscript. HZ, LW, ZM, Y’aC, and HF analyzed the data, read the manuscript, and advised on method development. All authors have approved the final version of the manuscript. All authors contributed to the article.
